# Supplementary figures and images for: ATM-Mediated translocation of RanBPM regulates DNA damage response by stabilizing p21 in non-small cell lung cancer cells
Source: Cell Oncol (Dordr). 2023 Sep 7;47(1):245–58. doi: 10.1007/s13402-023-00866-x (PMC10899406; doi:10.1007/s13402-023-00866-x)

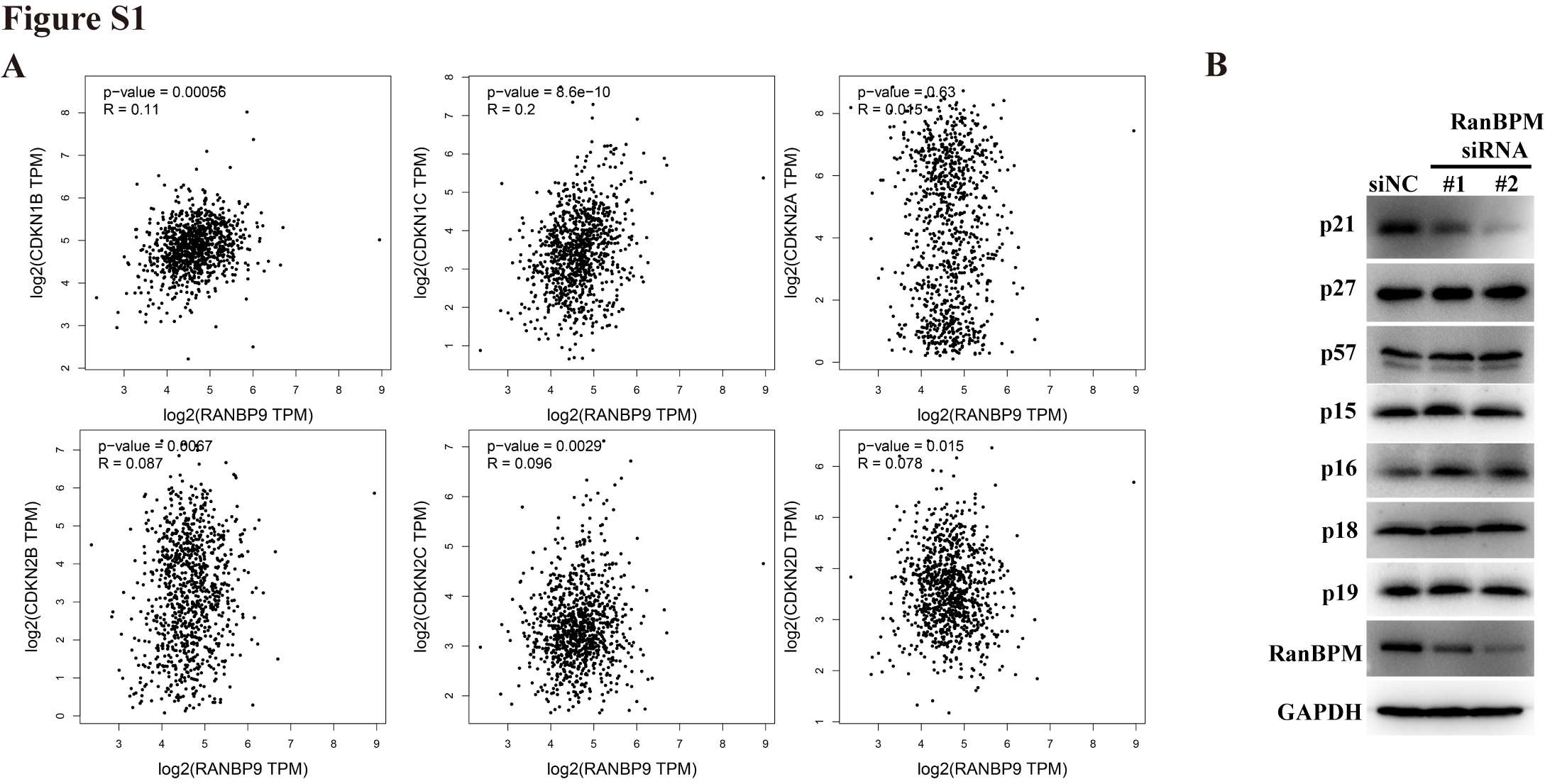

Supplement: Supplementary file 1 — Supplementary Material 1: Figure S1. (A) Correlation analysis of the mRNA levels of RanBPM and CKIs genes in NSCLC samples from TCGA datasets. (B) A549 cells were transiently transfected with the indicated siRNAs, and the proteins were assessed by western blotting. [file 13402_2023_866_MOESM1_ESM.png]

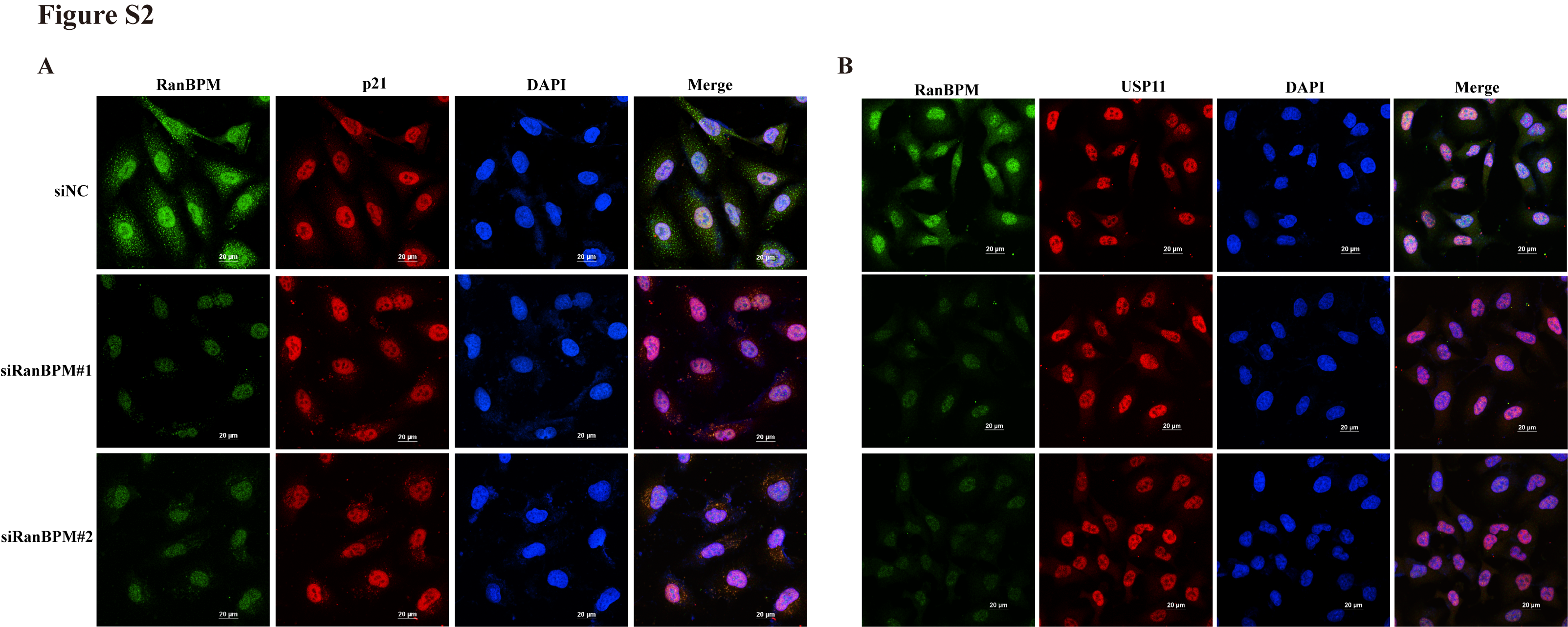

Supplement: Supplementary file 2 — Supplementary Material 2: Figure S2. RanBPM does not regulate the subcellular location of p21 and USP11. (A and B) A549 cells transfected with the indicated siRNA were treated with MG132 (20 μM) for 6 h, then were fixed and stained. DAPI was used for nuclei staining. Scale bars represent 20 μm. [file 13402_2023_866_MOESM2_ESM.png]
